# Predicting clinically actionable metagenomic next-generation sequencing in sepsis through dynamic inflammatory trajectories

**DOI:** 10.3389/fmicb.2026.1949602

**Published:** 2026-10-20

**Authors:** Xiaocheng Ding, Peng Bao, Xiaopeng Huang, Rui Xu, Xinyi Tang, Yuzhi Wu, Xiaoli Cui, Yang Lu

**Affiliations:** Department of Intensive Care Medicine, Affiliated Hospital of Nantong University, Nantong, Jiangsu, China

**Keywords:** antimicrobial stewardship, clinical actionability, clinical prediction model, dynamic inflammatory trajectory, metagenomic next-generation sequencing, sepsis

## Abstract

**Background:**

Metagenomic next-generation sequencing (mNGS) improves pathogen detection in sepsis, but positive results do not always prompt antimicrobial modification. We investigated whether dynamic host-response trajectories during the mNGS peri-report interval could predict clinical actionability.

**Methods:**

This retrospective single-center study included 109 adults with sepsis who underwent mNGS and conventional culture, and 72 patients with complete prespecified predictor data entered model development. Baseline and dynamic logistic models were assessed for discrimination, calibration, prediction error, and exploratory decision-curve performance. Final fixed models underwent 1,000-resample bootstrap optimism correction.

**Results:**

mNGS positivity was 94.8% in the actionable group and 84.3% in the non-actionable group, with broader descriptive pathogen detection than culture. In the complete-case cohort, the peri-report change in white blood cell count (dWBC) remained associated with actionability after adjustment for the included baseline variables (adjusted OR, 1.14; 95% CI, 1.02–1.28; *P* = 0.025). Apparent AUC increased from 0.744 to 0.816 after adding dWBC. Optimism-corrected AUCs were 0.697 and 0.768, and corrected Brier scores were 0.228 and 0.207. No baseline-WBC interaction was detected (*P* = 0.494).

**Conclusion:**

A less pronounced decline or an increase in WBC from pre-mNGS sampling to post-report reassessment was associated with a greater likelihood of subsequent documented mNGS-related antimicrobial modification.

## 1 Introduction

Sepsis is a life-threatening syndrome characterized by organ dysfunction due to dysregulated host response to infection, and it remains one of the leading causes of death in critically ill patients despite significant advances in intensive care and organ support technologies (Singer et al., 2016). According to the Global Burden of Disease Study, approximately 49 million people suffer from sepsis annually, resulting in nearly 11 million deaths worldwide and accounting for nearly one-fifth of the total global deaths (Rudd et al., 2020). Early administration of appropriate antibacterial treatment is a central determinant of outcome, whereas delays in effective treatment are associated with adverse outcomes (Bloos et al., 2017; Evans et al., 2021; Kadri et al., 2021; Kumar et al., 2006; Rhee et al., 2020). However, due to the inability to obtain pathogen information during the initial clinical assessment, empirical broad-spectrum antibiotic treatment remains the core of early sepsis management, which often leads to inappropriate use of antibacterial drugs or delayed optimization of treatment. Traditional microbial culture remains fundamental for pathogen identification and susceptibility testing but is limited by prolonged turnaround, reduced sensitivity after prior antimicrobial treatment, and poor recovery of fastidious or non-culturable organisms (Chiu and Miller, 2019; Gu et al., 2019; Simner et al., 2018). Rapid pathogen detection is therefore an important component of antimicrobial stewardship in sepsis.

Metagenomic next-generation sequencing (mNGS) is a culture-independent technology approach capable of detecting a broad range of bacteria, fungi, DNA and RNA viruses, and parasites directly from a single clinical sample without prior knowledge of the suspected pathogen (Zhao et al., 2024). Compared with culture, mNGS can provide broader pathogen coverage and higher sensitivity in selected infectious syndromes, including sepsis and diagnostically challenging central nervous system infections (Benoit et al., 2024; Wilson et al., 2019; Xu et al., 2025). However, improved detection does not necessarily translate into a change in management. Most previous studies mainly focused on diagnostic performance, including positive rates, pathogen spectra, and diagnostic accuracy, and fewer studies explored whether mNGS results ultimately led to clinically significant adjustments in antimicrobial drugs. Recent studies have emphasized that when evaluating the clinical value of mNGS, not only their diagnostic effects should be considered, but also their ability to guide the optimization of antibacterial drugs and improve patient treatment (Daher et al., 2024; Zhang et al., 2024).

Moreover, in the practice of routine ICU, a positive mNGS test result does not necessarily mean that the antibiotic treatment needs to be adjusted. The detected microorganisms could represent colonization, contamination, or microorganisms that have already been adequately covered by empirical treatment, implying that additional microbial information does not always change clinical management. Therefore, the clinical value of mNGS not only depends on the detection result of the pathogen itself, but also on the changes in the condition of patient and the specific context when interpreting the results (Zhao et al., 2024).

In recent years, sepsis has increasingly been recognized as a highly heterogeneous syndrome, evolving syndrome in which longitudinal host-response measures may provide information beyond a single baseline value (Kox et al., 2026; Seymour et al., 2019; Stanski and Wong, 2020). Compared with a single baseline measurement, the longitudinal changes in inflammatory markers may more accurately reflect disease progression and treatment response, thereby providing additional information for individualized clinical decision-making. These observations suggest that the dynamic trajectory of the host response may also influence the clinical utility of microbiological diagnostic tests such as mNGS. At the same time, predictive modeling, as a method for identifying patient subgroups most likely to benefit from specific diagnostic or therapeutic interventions, has also received increasing research exploration, which could help to characterize the probability that newly available diagnostic information will be translated into a clinical action at a defined decision point (Stanski and Wong, 2020).

We hypothesized that changes in routinely available inflammatory indicators between the pre-mNGS assessment and post-report clinical reassessment could provide additional context for interpreting available mNGS findings. We therefore developed a baseline model and a dynamic model incorporating peri-report inflammatory changes to predict subsequent mNGS-associated antimicrobial modification in patients with sepsis. This study not only focuses on diagnostic performance but also aims to explore whether dynamic host-response trajectories can enhance the predictive ability of mNGS for clinical actionability and conduct internal evaluation of the obtained models through the bootstrap resampling method.

## 2 Materials and methods

### 2.1 Study design and patient cohort

This retrospective single-center cohort was conducted in the Department of Intensive Care Medicine, Affiliated Hospital of Nantong University. The screening frame comprised consecutive adults with Sepsis-3-defined sepsis who underwent clinician-directed mNGS from January 2022 to November 2025 and it did not represent all ICU admissions. mNGS was ordered when broad, rapid pathogen investigation was considered necessary for severe or diagnostically uncertain infection. Conventional cultures were obtained from clinically relevant sites as routine care, and mNGS was used as an adjunct rather than a replacement.

Sepsis was defined according to the Sepsis-3 criteria as suspected or confirmed infection accompanied by an acute increase of ≥2 points in the Sequential Organ Failure Assessment (SOFA) score from baseline. Inclusion criteria were: (1) age ≥18 years; (2) diagnosed with sepsis according to Sepsis-3 criteria; and (3) availability of both mNGS and relevant conventional microbiological culture results during the same diagnostic episode. Patients were excluded from the overall analysis if essential baseline clinical information required for cohort characterization and outcome assessment was unavailable. Patients with incomplete mNGS or conventional microbiological culture records were also excluded where these data were required for the predefined analysis.

Of the 251 patients screened, 142 were excluded before the overall analysis due to incomplete key baseline clinical data. The remaining 109 patients constituted the overall analysis cohort, and patients were subsequently categorized into a clinically actionable group (n = 58) and a non-actionable group (n = 51) based on whether mNGS findings resulted in subsequent modification of antimicrobial therapy (Figure 1). Regarding model construction, complete data for all candidate predictors were required prior to univariate screening and LASSO selection. Among the 109 patients, 37 were excluded because dLnCRP could not be calculated due to missing post-report CRP data. The final cohort of the complete data model consisted of 72 patients, including 39 patients who received antimicrobial adjustments based on mNGS results and 33 patients who did not receive such adjustments.

**FIGURE 1:**
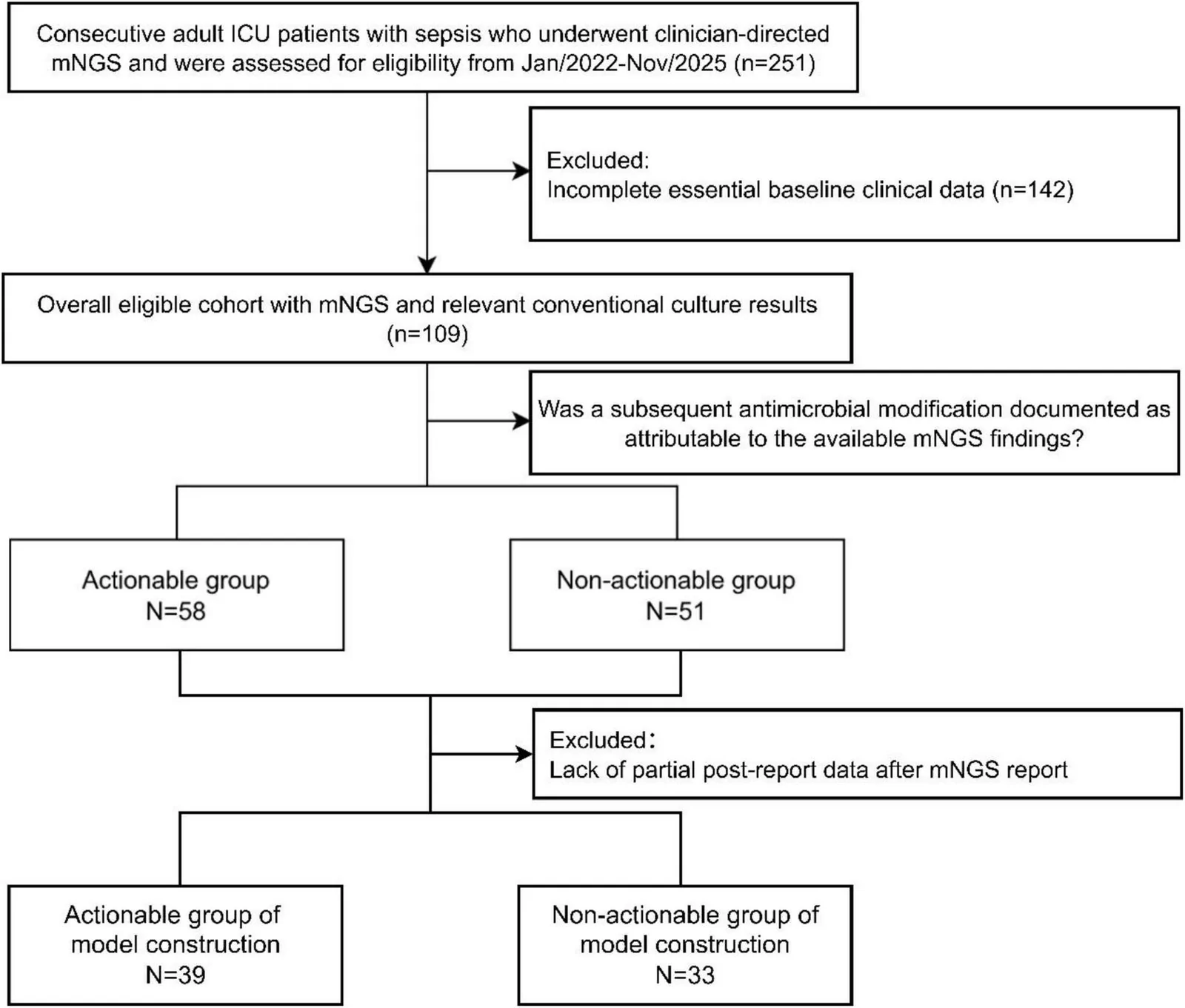
Study flowchart of patient selection. A total of 251 patients were screened, of whom 142 were excluded before the overall analysis because essential baseline clinical data was incomplete. The remaining 109 patients constituted the overall analytic cohort. For model development, 37 additional patients were excluded because dLnCRP could not be calculated owing to missing post-report CRP measurements. The final complete-case model-development cohort comprised 72 patients.

The study protocol was approved by the Ethics Committee of the Affiliated Hospital of Nantong University (Approval No. 2025-K266). Given the retrospective study design and the use of de-identified clinical data, the requirement for informed consent was waived by the Ethics Committee. The study was conducted in accordance with the principles of the Declaration of Helsinki.

### 2.2 Definition of clinically actionable mNGS results

The primary outcome was subsequent antimicrobial modification documented as associated with the available mNGS findings. It was a pragmatic measure of actionability and was not interpreted as treatment appropriateness, clinical benefit, or improved outcome.

Baseline measurements were obtained before or closest to mNGS specimen collection. The mNGS report then became available, followed by the first eligible repeat measurements; outcome-defining antimicrobial modification occurred only after both the report and repeat measurements. The post-report assessment window extended from report availability to discharge from the index hospitalization. Individual laboratory results were available to clinicians, but retrospectively constructed changes and model probabilities were not used clinically.

The primary team’s contemporaneous records provided the final diagnosis, antimicrobial changes, and documented rationale. Two experienced physicians from the same ICU team, neither of whom had directly cared for the included patients during the relevant admission, independently applied the prespecified actionability criteria; disagreements were resolved by consensus. Their role was to standardize classification, not to replace the treating team’s judgment.

Detection alone was insufficient for actionability. Adjudication considered the anatomical sampling site, clinical syndrome, imaging, conventional microbiology, treatment history, and biological plausibility of the organism. Each actionable case was assigned to one mutually exclusive primary category: initiation, escalation, de-escalation, discontinuation, antifungal modification, or antiviral modification; definitions and precedence rules are provided in Supplementary Table 1.

Non-actionable cases comprised patients whose therapy remained unchanged and those with a modification not documented as attributable to mNGS. This label did not imply absence of infection or absence of diagnostic, infection-control, prognostic, or public-health value. Broader non-pharmacological management effects were not systematically captured, and no counterfactual assessment was possible.

### 2.3 Conventional microbiological culture

All the enrolled patients underwent routine clinical care and received routine microbial cultures. According to the suspected source of infection, clinical specimens from the affected areas were collected using standard aseptic techniques, including blood, sputum, bronchoalveolar lavage fluid (BALF), and other specimens.

Pathogen isolation, identification, and antimicrobial susceptibility testing were performed in the clinical microbiology laboratory according to Clinical and Laboratory Standards Institute (CLSI) recommendations. Culture results were interpreted with the clinical context. mNGS and conventional culture were performed during the same diagnostic episode, although identical specimen types were not available for both methods in every patient; therefore, comparisons were conducted at the patient level.

### 2.4 Metagenomic next-generation sequencing testing

mNGS was performed by Dinfectome (Nanjing, China). Specimen types included blood, bronchoalveolar lavage fluid (BALF), sputum, cerebrospinal fluid, urine, and other body fluids. Specific types are reported descriptively in Table 1. For formal comparison, specimens were grouped as respiratory and non-respiratory because several individual categories were sparse. Specimens were collected aseptically. Microbial DNA was extracted using the TIANamp Magnetic DNA Kit (Tiangen Biotech, Beijing, China), and RNA using the RNA Virus Extraction Kit 1405005001 (Dinfectome Inc.). Quantity and quality were assessed with Qubit and NanoDrop instruments (Thermo Fisher Scientific).

**TABLE 1:** Baseline characteristics of the overall eligible cohort.

	Actionable group (N = 58)	Non-actionable group (N = 51)	*P* value
Age, years (median, IQR)	67[57, 78.75]	65[57.5, 72.5]	0.179
mNGS specimen category (n, %)			P < 0.001
Respiratory specimens	46 (79.31%)	23 (45.10%)	
BALF	29 (50.00%)	16 (31.37%)	
Sputum	17 (29.31%)	7 (13.73%)	
Non-respiratory specimens	12 (20.69%)	28 (54.90%)	
Blood	8 (13.79%)	24 (47.06%)	
Cerebrospinal fluid	2 (3.45%)	2 (3.92%)	
Urine	1 (1.72%)	1 (1.96%)	
Other body fluids	1 (1.72%)	1 (1.96%)	
Male (n, %)	43 (74.14%)	31 (60.78%)	0.199
Female (n, %)	15 (25.86%)	20 (39.22%)	0.199
Hypertension (n, %)	27 (46.55%)	20 (39.22%)	0.563
Diabetes (n, %)	11 (18.97%)	17 (33.33%)	0.135
CHD (n, %)	6 (10.34%)	5 (9.80%)	1.000
COPD (n, %)	1 (1.72%)	0 (0.00%)	1.000
SOFA (median, IQR)	7.50 [5.00, 12.00]	9.00 [6.00, 12.00]	0.301
APACHE II (median, IQR)	21.00 [13.00, 27.00]	20.00 [16.50, 27.50]	0.745
WBC, × 10^9^/L (median, IQR)	10.75 [7.28, 13.38]	13.20 [7.65, 18.68]	0.158
NEUT, %(median, IQR)	90.40 [83.78, 93.10]	88.60 [82.75, 93.10]	0.566
CRP, mg/L (median, IQR)	93.20 [37.84, 206.46]	118.90 [51.71, 210.74]	0.866
PCT, ng/mL (median, IQR)	1.95 [0.38, 14.00]	4.30 [1.31, 27.00]	0.051
Septic shock (n, %)	15 (25.86%)	14 (27.45%)	1.000
Mechanical ventilation (n, %)	47 (81.0%)	39 (76.5%)	0.728
Mechanical ventilation days (median, IQR)	12.00 [3.00, 23.00]	6.00 [1.00, 13.50]	0.075
Length of hospital stay (median, IQR)	21.00 [13.00, 33.75]	18.00 [9.00, 28.50]	0.112
Length of ICU stay (median, IQR)	17.00 [8.50, 28.50]	13.00 [8.00, 24.50]	0.230
Antimicrobial exposure before mNGS, days (median, IQR)	3.00 [2.00, 5.00]	4.00 [2.00, 8.50]	0.517
Total hospitalization cost, × 10^4^ CNY (median, IQR)	16.95 [9.61, 28.14]	10.41 [6.80, 27.00]	0.104

Data are median [IQR] or n (%). Total hospitalization cost refers to all expenditures during the index admission and not the mNGS assay cost (values appear consistent with × 10^4^ CNY). For specimen category, the *P* value compares respiratory with non-respiratory specimens using Pearson’s Chi-square test, and individual specimen types are presented descriptively. BALF, bronchoalveolar lavage fluid; CHD, coronary heart disease; COPD, chronic obstructive pulmonary disease; SOFA, sequential organ failure assessment; APACHE II, acute physiology and chronic health evaluation II; WBC, white blood cell count; NEUT, neutrophil percentage; CRP, C-reactive protein; PCT, procalcitonin; ICU, intensive care unit.

DNA libraries were prepared using the Hieff NGS C130P2 OnePot II DNA Library Prep Kit for MGI (Yeasen Biotechnology, Shanghai, China). RNA libraries were prepared using the VAHTS Universal V8 RNA-seq Library Prep Kit for MGI (Vazyme, Nanjing, China). Library quality was assessed using the Agilent 2100 Bioanalyzer (Agilent Technologies, Santa Clara, CA, USA) and Qubit 4.0 fluorometer (Thermo Fisher Scientific, Waltham, MA, USA). Qualified libraries were sequenced as 50-bp single-end reads on the DIFSEQ-200 platform (Dinfectome Inc., Nanjing, China).

Raw data were processed using a standardized pipeline. After demultiplexing with bcl2fastq2 (version 2.20), adapter sequences, duplicate reads, low-quality reads, and reads shorter than 36 bp were removed using Trimmomatic (version 0.36). Human reads were subtracted using Bowtie2 (version 2.2.6) against hs37d5. Remaining reads were taxonomically classified using Kraken2 (version 2.0.7) with a custom GenBank-based microbial reference database (version 238, ftp://ftp.ncbi.nlm.nih.gov/genomes/genbank/), and their abundance was estimated using Bracken (version 2.5.0). The reference database contains genomic sequences of bacteria, fungi, viruses, and parasites. Laboratory findings were subsequently interpreted together with the clinical context when actionability was adjudicated.

### 2.5 Clinical data collection and construction of dynamic variables

Clinical data were retrospectively extracted from the electronic medical record system. Baseline variables included demographic characteristics (age and gender), comorbidities (hypertension, diabetes, coronary artery disease, and chronic obstructive pulmonary disease), disease severity indicators upon ICU admission, such as the Acute Physiology and Chronic Health Evaluation II (APACHE II) score and Sequential Organ Failure Assessment (SOFA) score, as well as basic laboratory parameters, including white blood cell (WBC) count, neutrophil percentage (NEUT), C-reactive protein (CRP), and procalcitonin (PCT). Clinical management and outcome variables were also collected, including septic shock, mechanical ventilation, mechanical ventilation duration, ICU stay duration, total hospital stay duration, duration of antibiotic therapy, and hospitalization cost.

Empirical antimicrobial therapy was generally initiated before the mNGS report became available. For construction of dynamic variables, the baseline value was defined as the measurement obtained before or closest to mNGS specimen collection. The follow-up value was defined as the first available measurement obtained after mNGS report availability but before any subsequent antimicrobial modification classified as the study outcome. Measurement timing followed routine clinical practice and was not standardized. The exact interval between mNGS report availability and repeat laboratory measurement could not be reliably reconstructed for all patients and was therefore neither summarized nor included as a model predictor.

The dynamic variables were calculated as follows:


$$\text{dX}={\text{X}}_{\mathrm{post}-\mathrm{report}}-{\text{X}}_{\text{baseline}}$$

where X_*baseline*_ represented the pre-mNGS value and X_*post–report*_ represented the post-report value obtained before antimicrobial modification. Therefore, all dynamic predictors preceded the study outcome, although the individual follow-up laboratory results were available to the treating clinicians when treatment decisions were made. These variables describe the change from pre-mNGS assessment to post-report reassessment and are therefore referred to as peri-report dynamic changes.

The prespecified candidate dynamic variables included changes in white blood cell count (dWBC), neutrophil percentage (dNEUT), log-transformed PCT (dLnPCT), log-transformed CRP (dLnCRP), SOFA score (dSOFA), and APACHE II score (dAPACHE II) during the mNGS peri-report interval. All candidate dynamic variables were evaluated using the same model-development procedure.

### 2.6 Model development

The baseline model used variables available before mNGS testing, whereas the dynamic model additionally incorporated changes available during post-report reassessment but before antimicrobial modification. The dynamic model was therefore developed for post-report use and was not intended to guide the decision to order mNGS.

Candidate predictors were identified prior to statistical screening. Within the total analysis cohort of 109 patients, the extent of missing data was assessed before univariate analysis and LASSO screening to ensure that all candidate predictors were evaluated within the same model-building population. Of these, 37 patients lacked post-report CRP data, rendering the calculation of dLnCRP impossible. The remaining 72 patients had complete data for all candidate predictors, forming the complete-case cohort used for model construction (Supplementary Table 2).

Candidate predictors were initially screened using univariable logistic regression. Variables with a two-sided *P* < 0.20 were eligible for subsequent LASSO logistic regression with five-fold cross-validation. Variables retained by LASSO were entered into the final unpenalized multivariable logistic regression models. Model development was performed in the 72-patient complete-case cohort without imputation, and the final models were fitted using predictors on their original measurement scales. Odds ratios, 95% confidence intervals, and two-sided *P* values were reported.

Because post-report CRP was unavailable in 37 patients, the complete candidate-selection procedure could not be repeated in the overall cohort. As other predictors retained in the finalized dynamic model were available for all 109 patients, we performed two *post hoc* sensitivity analyses: univariable analysis of key predictors and refitting of the finalized predictor set without repeating variable selection.

### 2.7 Model performance and bootstrap optimism assessment

Predicted probabilities were calculated from the final multivariable models. Discrimination was assessed by AUC, prediction error by Brier score, and calibration by intercept, slope, and plots. Apparent decision-curve performance was explored using DCA.

An exploratory threshold analysis was performed for feature predictors using the Youden index. Because the cutoff was derived from the same model-development cohort, it was considered descriptive and was not proposed for clinical implementation without independent validation.

Conditional bootstrap optimism assessment used 1,000 nonparametric resamples of the 72-patient complete-case cohort. In each resample, the finalized models were refitted with the same predictors and evaluated in both the resample and the original cohort. Corrected AUC was calculated by subtracting mean AUC optimism from the apparent AUC, whereas corrected Brier score was calculated by adding mean Brier-score degradation. Corrected calibration intercepts and slopes summarized application of the bootstrap-refitted models to the original cohort, with percentile 95% confidence intervals. Univariable screening and LASSO selection were not repeated. Therefore, these estimates are conditional on the finalized predictor sets.

### 2.8 Baseline subgroup and interaction analyses

Exploratory analysis was conducted to assess whether baseline indicators would affect the association between dynamic indicators and clinical actionability. Patients were stratified based on clinically interpretable baseline thresholds. In each stratum, a multivariate logistic regression model was used to adjust the estimation of the association between dynamic indicators and actionability. Since the baseline indicators had already defined the strata, they were not reused as covariates.

The effect modification was formally evaluated in the complete patient model development cohort through the product interaction term between consecutive baselines and consecutive indicators. Both variables were mean centered before constructing the interaction term. The interaction was tested by comparing the likelihood ratios of models with and without the interaction term.

### 2.9 Composite clinical reference standard

The same two non-treating critical care physicians independently assigned the composite etiological classification after reviewing clinical presentation, imaging, laboratory findings, conventional microbiology, antimicrobial exposure, treatment response, and all pathogen-testing results. Disagreements were resolved by consensus. Because mNGS contributed to this composite assessment, the reference standard was not independent of the index test.

For each microbiological test method, a true positive result was defined as the detection of at least one microorganism that was considered to cause or potentially cause the clinical infection syndrome. Detection of colonizing, contaminating, or clinically irrelevant microorganisms was classified as a false positive result. A false negative result was defined as the failure to detect the pathogen based on the composite clinical reference criteria, while a true negative result required a negative test result and the absence of a clearly identified pathogenic microorganism.

### 2.10 Statistical analysis

Continuous variables are summarized as median (IQR) and categorical variables as n (%). Groups were compared using the Mann-Whitney U test, Chi-square test, or Fisher exact test, as appropriate. Predictor-level missingness in the 109-patient cohort is reported in Supplementary Table 2. The 72 complete cases and 37 modeling exclusions were compared using hypothesis tests and standardized mean differences (Supplementary Table 3).

Sensitivity, specificity, predictive values, and accuracy were calculated against the composite clinical assessment and interpreted as exploratory concordance measures because the reference standard incorporated mNGS.

Apparent AUCs from the paired predictions of the baseline and dynamic models were compared using DeLong’s test. DCA used apparent predictions and was treated as exploratory rather than evidence of established clinical utility. Bootstrap analyses used a fixed random seed, and successful and failed resamples were recorded.

Analyses were conducted in Python 3 (Python Software Foundation, Wilmington, Delaware, USA) using pandas, NumPy, SciPy, scikit-learn, and statsmodels. The bootstrap optimism assessment used a fixed random seed of 202607. All statistical tests were two-sided, and *P* < 0.05 was considered statistically significant.

## 3 Results

### 3.1 Study population and baseline characteristics

Among 251 mNGS-tested patients screened, 142 were excluded because essential baseline data were incomplete. The overall cohort comprised 109 patients (58 actionable and 51 non-actionable), 72 complete cases entered model development, whereas 37 lacked at least one paired CRP measurement required for dLnCRP (Figure 1 and Supplementary Table 2).

Actionability rates and dWBC distributions were similar in the complete-case and excluded cohorts (54.2% vs. 51.4%, *P* = 0.841; dWBC *P* = 0.833). Male sex was more frequent among complete cases (88.9% vs. 27.0%, *P* < 0.001; SMD = 1.253); hospitalization cost and dSOFA also differed (Supplementary Table 3).

Among 58 actionable cases, the mutually exclusive primary modifications were initiation in 0 (0.0%), escalation in 39 (67.2%), de-escalation in 8 (13.8%), discontinuation in 1 (1.7%), antifungal modification in 8 (13.8%), and antiviral modification in 2 (3.4%) patients (Supplementary Table 1).

Among 51 non-actionable cases, antimicrobial therapy remained unchanged in 49 (96.08%), whereas 2 (3.92%) underwent modification not documented as attributable to mNGS (Supplementary Table 1).

Respiratory specimens were more frequent in the actionable group than in the non-actionable group (79.3% vs. 45.1%, *P* < 0.001). No statistically significant differences were observed in the other demographic, comorbidity, severity, inflammatory, or clinical-management characteristics reported in Table 1 (all *P* > 0.05).

### 3.2 Comparison of diagnostic performance between mNGS and conventional microbiological culture

At the patient level, mNGS detected at least one microorganism in 98/109 patients (89.9%), compared with 50/109 (45.9%) for conventional culture. Detection yields were 55/58 (94.8%) versus 31/58 (53.4%) in the actionable group and 43/51 (84.3%) versus 19/51 (37.3%) in the non-actionable group (Figure 2).

**FIGURE 2:**
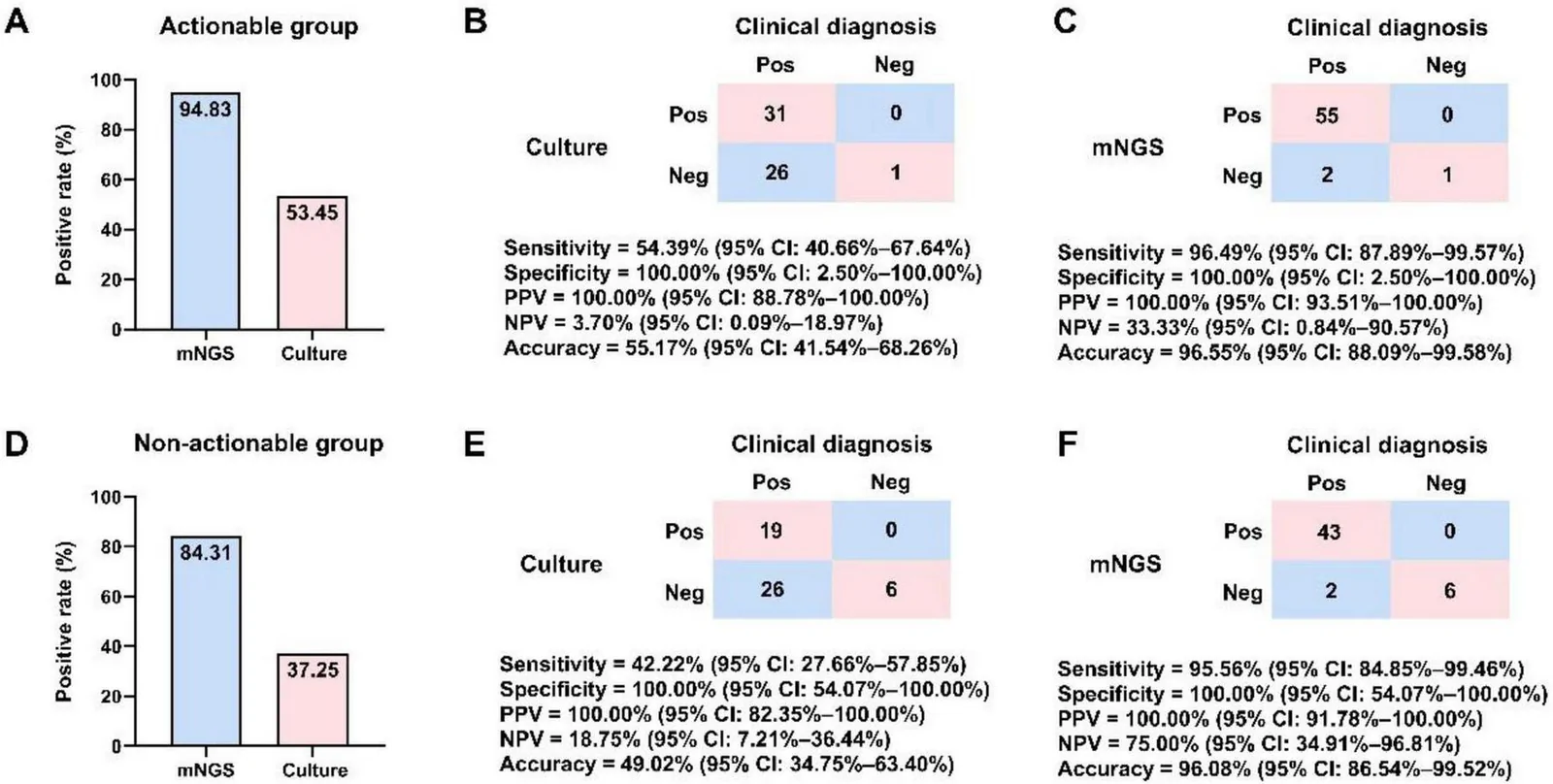
Patient-level detection yield and exploratory concordance with the composite clinical assessment. **(A–C)** Actionable group; **(D–F)** non-actionable group. Because the composite assessment incorporated mNGS and specimens were not uniformly matched, sensitivity, specificity, predictive values, and accuracy are exploratory concordance measures rather than independent diagnostic-accuracy estimates. PPV, positive predictive value; NPV, negative predictive value.

Exploratory concordance estimates against the composite clinical assessment favored mNGS; however, the assessment incorporated mNGS and specimen types were not uniformly matched. These estimates therefore do not establish independent diagnostic superiority.

High detection yield in both groups did not uniformly translate into documented antimicrobial modification, underscoring the distinction between detection and actionability.

### 3.3 Microbial spectrum identified by mNGS and conventional culture

mNGS showed a broader descriptive organism spectrum than conventional culture (Figure 3). In addition to a greater diversity of bacterial taxa, mNGS identified 12 fungal taxa and 18 DNA or RNA viral taxa that were not recovered by conventional culture. Descriptively, the actionable group showed a more diverse organism spectrum, including fungal, viral, and polymicrobial detections, whereas common bacterial organisms accounted for a larger share of detections in the non-actionable group. The higher microbial complexity in patient populations where clinical decision-making can be guided may increase the likelihood that mNGS provides clinically relevant information, thereby influencing antimicrobial selection decisions, but formal statistical testing is still needed to validate it as evidence of clinical benefit.

**FIGURE 3:**
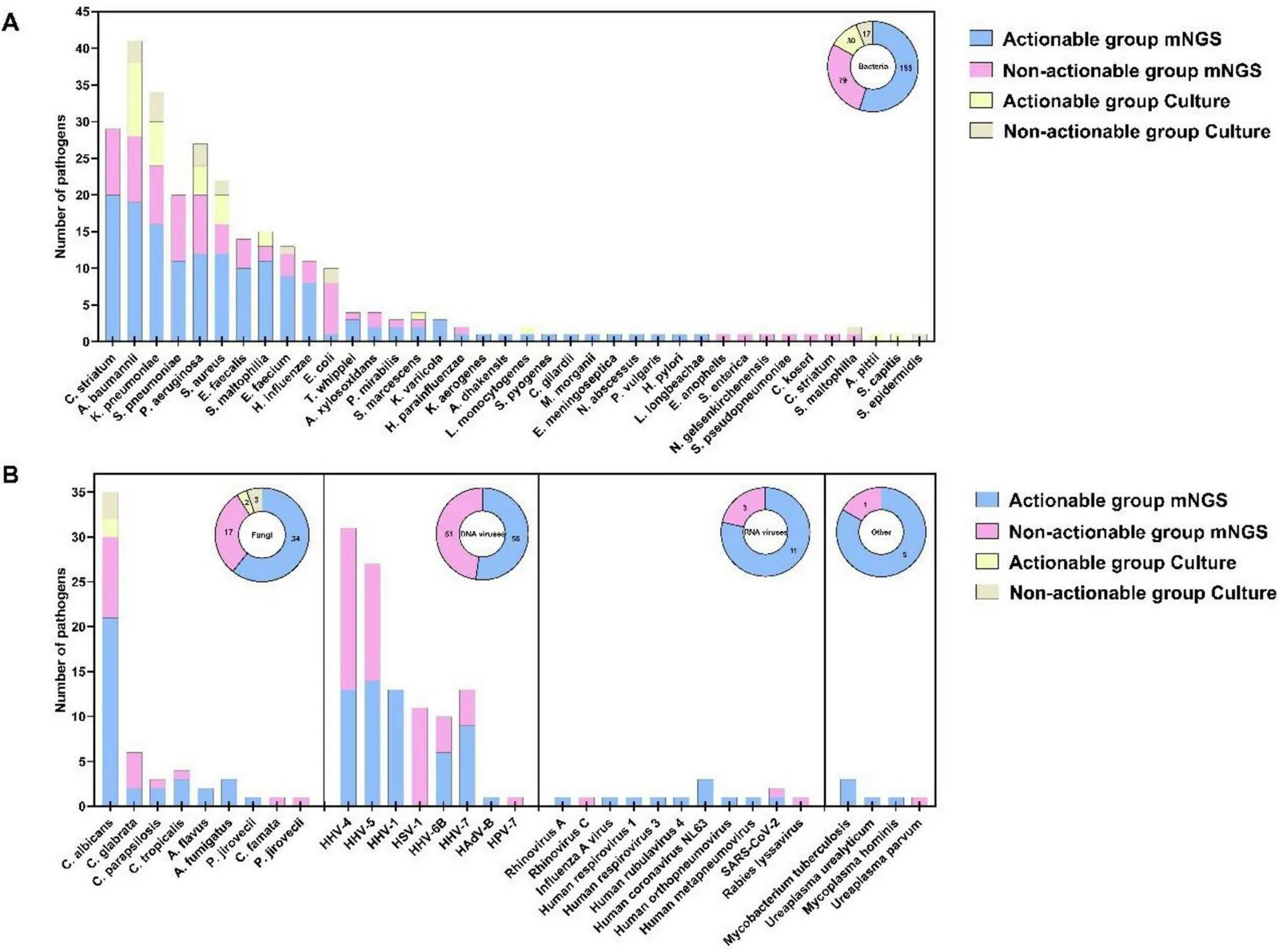
The microbial spectrum identified by mNGS and conventional culture. **(A)** Distribution of bacterial pathogens detected by mNGS and conventional culture in clinically actionable and non-actionable patients. The inset chart summarizes the total number of bacterial species identified in each subgroup. **(B)** Distribution of fungal, DNA viral, RNA viral, and other pathogens detected by mNGS and conventional culture. The inset charts summarize the overall pathogen counts within each microbial category. “Non-actionable” denotes absence of a qualifying documented mNGS-related antimicrobial modification and does not imply absence of diagnostic, prognostic, infection-control, or public-health relevance.

### 3.4 Identification of predictors for clinically actionable mNGS results

In the complete-case cohort, baseline values preceded mNGS testing and follow-up values were obtained after report availability but before outcome-defining antimicrobial modification; thus, all model predictors preceded the outcome.

Univariable results are summarized in Table 2. Higher baseline WBC (OR, 0.924; 95% CI, 0.866–0.986; *P* = 0.017) and ln(PCT) (OR, 0.749; 95% CI, 0.587–0.957; *P* = 0.021) were associated with a lower likelihood of subsequent documented mNGS-related antimicrobial modification. Age and diabetes met the prespecified *P* < 0.20 screening threshold.

**TABLE 2:** Univariable logistic regression analysis of baseline and dynamic variables associated with mNGS clinical actionability.

Variables	Baseline Model Univariable OR (95% CI)	*P*_value
Baseline predictors		
WBC_pre	0.924 (0.866–0.986)	0.017
LnPCT_pre	0.749 (0.587–0.957)	0.021
Diabetes	0.383 (0.130–1.130)	0.082
Age	1.023 (0.994–1.053)	0.130
Hypertension	1.429 (0.562–3.632)	0.454
Mechanical ventilation duration before mNGS, days	1.008 (0.987–1.029)	0.457
APACHEII_pre	1.022 (0.965–1.082)	0.462
Shock	0.684 (0.218–2.140)	0.514
LnCRP_pre	1.102 (0.766–1.584)	0.601
CHD	1.471 (0.324–6.679)	0.617
Respiratory specimen (vs non-respiratory)	1.273 (0.466–3.477)	0.638
Antibiotic_days_pre	0.987 (0.925–1.054)	0.704
Sex	1.207 (0.277–5.253)	0.802
NEUT_pct_pre	0.996 (0.958–1.036)	0.839
SOFA_pre	0.998 (0.892–1.117)	0.973
Dynamic predictors		
dWBC	1.126 (1.039–1.221)	0.004
dSOFA	1.091 (0.954–1.248)	0.202
dLnPCT	1.143 (0.909–1.437)	0.255
dNEUT	1.023 (0.976–1.073)	0.338
dAPACHEII	0.961 (0.878–1.052)	0.385
dLnCRP	1.006 (0.754–1.342)	0.969

Data are presented as odds ratios (ORs) with 95% confidence intervals (CIs). WBC, white blood cell count; NEUT, neutrophil percentage; CRP, C-reactive protein; PCT, procalcitonin; SOFA, Sequential Organ Failure Assessment; APACHE II, Acute Physiology and Chronic Health Evaluation II; CHD, coronary heart disease; OR, odds ratio; CI, confidence interval.

Among the six candidate dynamic indicators, dWBC was the only variable that met the prespecified univariable screening criterion (OR = 1.126, 95% CI: 1.039–1.221, *P* = 0.004). Changes in SOFA score, APACHE II score, neutrophil percentage, ln(CRP), and ln(PCT) did not meet the screening criterion. Following the subsequent LASSO-based selection procedure, dWBC was retained as the dynamic predictor for the final multivariable model.

These findings identified dWBC as the retained dynamic candidate for further modeling; they do not establish that dWBC causally influenced treatment decisions or patient outcomes.

### 3.5 Development and performance of the dynamic prediction model

Following the prespecified screening and LASSO-based selection procedure, two final unpenalized logistic regression models were fitted in the 72-patient complete-case cohort. The baseline model included age, diabetes, baseline WBC, and baseline ln(PCT), whereas the dynamic model additionally included dWBC, the only dynamic variable retained through the model-development procedure (Figures 4A, B). To provide complete model specifications, the fitted equations on the original measurement scales were:

**FIGURE 4:**
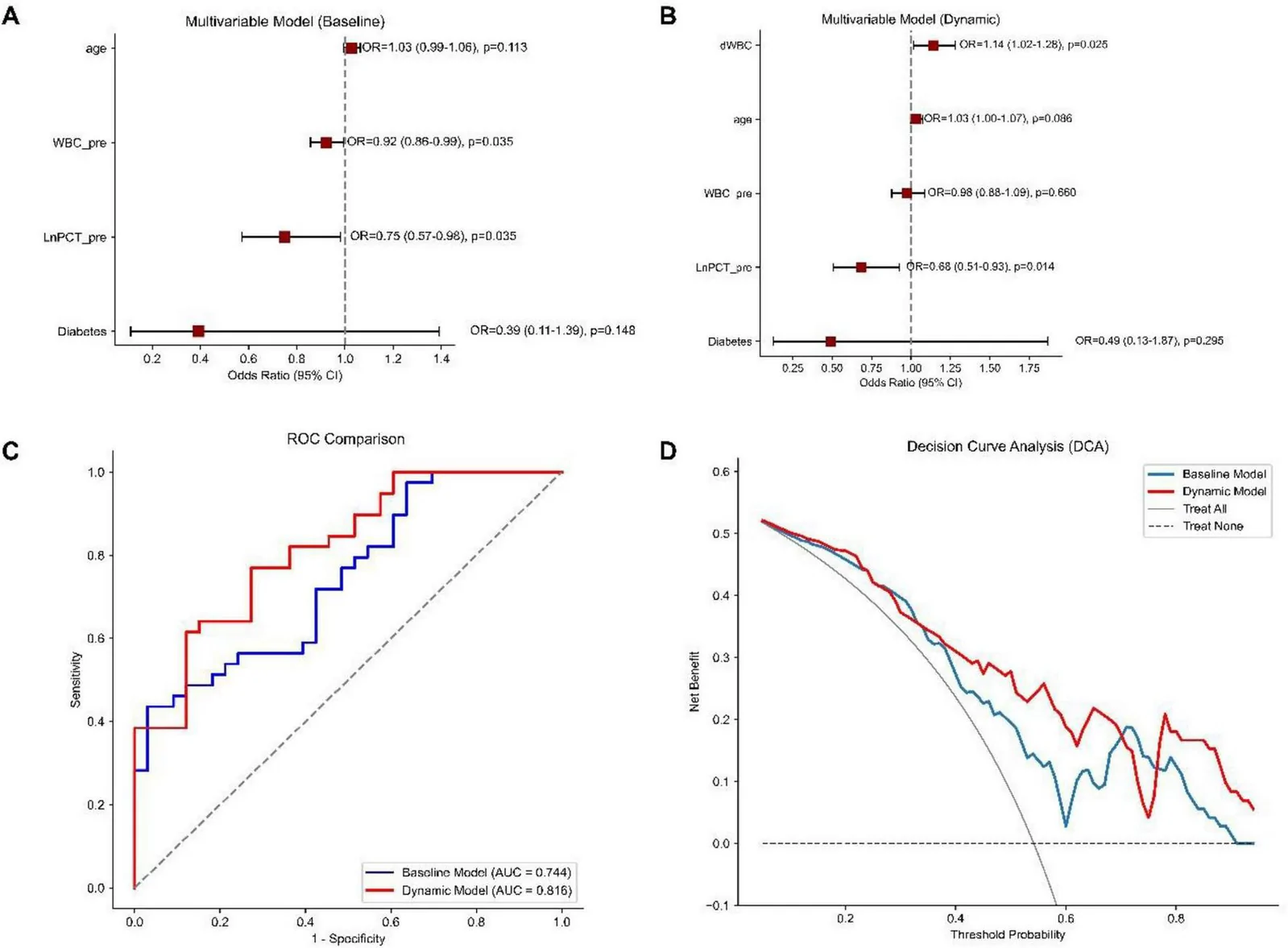
Development and performance evaluation of prediction models. Forest plot of the baseline multivariable logistic regression model **(A)** and dynamic multivariable logistic regression model **(B)** . **(C)** Receiver operating characteristic (ROC) curves comparing the discrimination performance of the baseline and dynamic models. **(D)** Decision-curve analysis (DCA) comparing the apparent decision-curve performance of the baseline and dynamic models. WBC, white blood cell count; PCT, procalcitonin; OR, odds ratio; CI, confidence interval; ROC, receiver operating characteristic; AUC, area under the curve; DCA, decision curve analysis.

Baseline model:

logit(p) = 0.013729+0.027645 × Age−0.934243 × Diabetes −0.079662 × WBCpre−0.289007 × ln(PCTpre).

Dynamic model:

logit(p) = −0.902909+0.033650 × Age−0.716613 × Diabetes −0.023768 × WBCpre−0.378983 × ln(PCTpre)+0.132499 × dWBC.

here, p denotes the model-estimated probability of a clinically actionable result. Diabetes was coded as 1 for presence and 0 for absence; age was expressed in years; WBCpre and dWBC were expressed in × 10^9^/L; and ln(PCTpre) represented the natural logarithm of PCT measured in ng/mL. Predicted probability was calculated as p = 1/[1+exp(−logit(p))].

Among all 109 patients with available dWBC, the association between dWBC and actionability remained positive in univariable analysis (OR, 1.05; 95% CI, 1.01–1.10; *P* = 0.025). In the *post hoc* fixed-predictor analysis, the dWBC coefficient remained positive but was attenuated after adjustment for age, diabetes, baseline WBC, and baseline ln (PCT) (adjusted OR, 1.06; 95% CI, 0.99–1.13; *P* = 0.092).

The apparent AUC was 0.744 (95% CI, 0.626–0.842) for the baseline model and 0.816 (95% CI, 0.708–0.904) for the dynamic model. The paired DeLong comparison did not reach statistical significance (*P* = 0.088). The corresponding apparent Brier scores were 0.199 and 0.173. Thus, the point estimates indicated numerically higher apparent discrimination and lower prediction error after adding dWBC (Figure 4C and Supplementary Figure 1).

Internal validation was conducted using 1000 bootstrap resamples, all of which were successfully fitted. The mean optimism in AUC was 0.046 for the baseline model and 0.048 for the dynamic model, resulting in optimism-corrected AUCs of 0.697 (95% CI, 0.598–0.820) and 0.768 (95% CI, 0.678–0.868), respectively. The optimism-corrected Brier scores were 0.228 for the baseline model and 0.207 for the dynamic model. The corrected calibration intercepts were 0.054 (95% CI, −0.461 to 0.533) and 0.076 (95% CI, −0.412 to 0.530), respectively. The corrected calibration slopes were 0.783 (95% CI, 0.442–1.306) for the baseline model and 0.732 (95% CI, 0.356–1.170) for the dynamic model (Supplementary Table 4 and Supplementary Figure 2). Thus, the dynamic model retained higher discrimination and lower prediction error after optimism correction, although the calibration slopes indicated some degree of overfitting. Exploratory DCA based on apparent predictions showed a higher estimated net benefit for the dynamic model across portions of the examined threshold range. This analysis was not optimism-corrected and does not establish validated clinical utility or patient benefit (Figure 4D).

### 3.6 Baseline-WBC subgroup and interaction analysis

In order to avoid the chance that this association merely reflected baseline inflammatory burden, we formally tested for effect modification by baseline WBC levels. Among patients with baseline WBC ≤ 12 × 10^9^/L, 25 of 37 had actionable results, and the adjusted OR for dWBC was 1.15 (95% CI 0.95–1.38, *P* = 0.144). Among patients with baseline WBC > 12 × 10^9^/L, 14 of 35 had actionable results, and the adjusted OR was 1.16 (95% CI 1.01–1.34, *P* = 0.034). Although statistical significance was reached only in the higher-WBC stratum, the point estimates were nearly identical and the confidence intervals overlapped (Supplementary Table 5 and Supplementary Figure 3).

The formal continuous interaction test was not significant (likelihood-ratio *P* for interaction = 0.494), providing no evidence that the association between dWBC and actionability differed according to baseline WBC. The subgroup finding should nevertheless be interpreted as exploratory because of the limited stratum-specific sample sizes.

### 3.7 Exploratory threshold analysis

An exploratory Youden-index analysis identified a dWBC cutoff of −6.81 × 10^9^/L, with an AUC of 0.714 (Figure 5A). The proportion of actionable results was 66.1% among patients with dWBC ≥ −6.81 × 10^9^/L and 12.5% among those below this cutoff (Figure 5B). Thus, a WBC decline of less than 6.81 × 10^9^/L or an increase was associated with a greater likelihood of subsequent documented mNGS-related antimicrobial modification.

**FIGURE 5:**
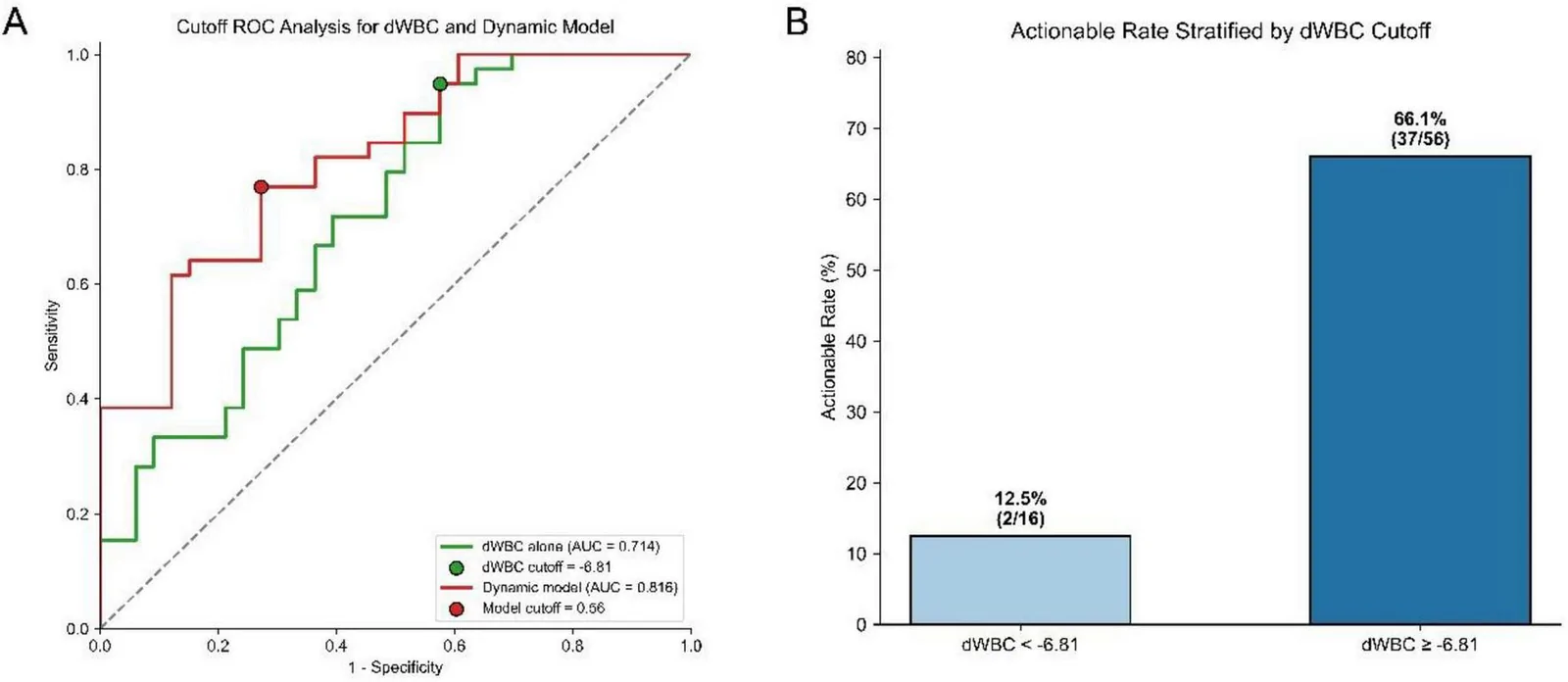
Exploratory threshold analysis of dWBC and the dynamic prediction model. **(A)** The optimal cut-off point was determined using the Youden index, and the ROC analysis of dWBC was conducted to predict clinically actionable mNGS results. **(B)** Comparison of the proportion of clinically actionable mNGS results in patients above and below the optimal cut-off point of dWBC. ROC, receiver operating characteristic; AUC, area under the curve.

The dynamic model had an apparent AUC of 0.816, and a Youden-derived predicted-probability cutoff of 0.56 provided the best sensitivity-specificity balance within the model-development cohort. Both cutoffs were derived from the same small cohort, and they do not indicate uncontrolled inflammation or provide instructions to modify treatment. This threshold is exploratory, derived from the model-building cohort, and requires further validation in clinical practice.

## 4 Discussion

In this retrospective sepsis cohort, mNGS had a higher descriptive patient-level detection yield and a broader organism spectrum than conventional culture, but pathogen detection did not uniformly result in antimicrobial modification (Duan et al., 2021; Sun et al., 2022; Torres et al., 2022). Among the six prespecified peri-report dynamic indicators, dWBC was the only variable retained through the model-development procedure. In the broader 109-patient cohort, dWBC remained positively associated with actionability in univariable analysis. When the finalized predictor set was refitted, the coefficient was attenuated and no longer statistically significant but remained positive. After adjustment for the included baseline variables, a less pronounced decline or an increase in WBC was associated with a greater likelihood of subsequent documented mNGS-related antimicrobial modification. The similar effect estimates across the two baseline-WBC strata and the nonsignificant interaction (*P* = 0.494) provided no evidence that this association differed according to baseline WBC.

The current research results are biologically reasonable, as sepsis is increasingly regarded as a highly heterogeneous syndrome characterized by dynamic immune responses rather than static inflammatory states (Kox et al., 2026; Seymour et al., 2019; Stanski and Wong, 2020; Torres et al., 2022; Van Nynatten et al., 2025). Recent studies have shown that the host inflammatory trajectory provides more clinically relevant information than a single baseline measurement, and can reflect the dynamic changes in immune dysregulation, treatment responses, and disease progression (Jazayeri et al., 2023; Von Rekowski et al., 2026). Similarly, Kox et al. emphasized that sepsis is a continuous evolving process with obvious spatiotemporal variability in host response (Kox et al., 2026). Stanski and Wong further proposed the concept of predictive enrichment, highlighting the importance of identifying biologically differentiated patient subgroups that are most likely to benefit from specific interventions (Stanski and Wong, 2020; Van Nynatten et al., 2025). Within this framework, short-term WBC trajectories may reflect the combined effects of infection progression, empirical antimicrobial coverage, source control, and host immune status. For example, inadequate empirical coverage may contribute both to persistent or increasing WBC and to a greater likelihood of subsequent antimicrobial modification, whereas effective empirical therapy may be accompanied by declining WBC and reduce the additional influence of newly available mNGS findings. Previous antimicrobial exposure, corticosteroid treatment, immunosuppression, and delayed source control may similarly affect both WBC trajectories and clinical decisions. Accordingly, dWBC should be interpreted as an integrative signal within the observed clinical workflow, not as a causal effect or evidence of incremental benefit attributable specifically to mNGS. This interpretation is consistent with emerging approaches that integrate pathogen detection with dynamic host-response information (Kalantar et al., 2022; Langelier et al., 2018).

The other inflammatory indicators provided less evident predictive information for this endpoint. Baseline WBC and ln(PCT) were inversely associated with actionability, which may partly reflect broader empirical antimicrobial coverage among patients with greater initial inflammatory burden, although the present data cannot establish this mechanism. Short-term changes in CRP and PCT did not show clear associations with subsequent mNGS-related antimicrobial modification. However, statistical nonsignificance should not be interpreted as evidence that these biomarkers are generally unhelpful for infection assessment or treatment monitoring. Neutrophil percentage showed limited variability, while paired absolute neutrophil and lymphocyte counts and the neutrophil-to-lymphocyte ratio were not systematically available. Prospective studies could incorporate transcriptomic, proteomic, cytokine, metabolomic, or immune-cell signatures to provide a more comprehensive characterization of the host response (Blauwkamp et al., 2019; Kalantar et al., 2022; Langelier et al., 2018; Shankar-Hari et al., 2016). Dynamic indicators may be more informative than static measurements for identifying clinically actionable mNGS results (Sun et al., 2025), and longitudinal biomarkers with interpretable prediction models may support real-time assessment in critically ill patients (Fleuren et al., 2020; Jazayeri et al., 2023; Jiang et al., 2023).

Anatomical sampling sites may influence both the clinical interpretation of detected organisms and the likelihood that mNGS findings lead to antimicrobial modification. Respiratory specimens were more frequent in the actionable group than in the non-actionable group in the overall cohort (79.3% vs. 45.1%, *P* < 0.001). Specimen type may therefore have confounded the observed association between dWBC and actionability. Given the small model-development cohort and sparse individual specimen categories, its independent contribution could not be adequately estimated, and residual confounding cannot be excluded. Because the same organism may have different implications in respiratory and normally sterile-site specimens, anatomical and clinical context should be incorporated into post-report interpretation. Prospective studies with sufficient representation of individual specimen types are needed to clarify their independent contribution.

The predominance of respiratory specimens also places our findings in the context of rapid molecular testing for respiratory infection. A randomized trial in a pediatric emergency setting found that multiplex PCR point-of-care testing did not reduce overall antibiotic prescribing, although pathogen-targeted therapy was initiated in some patients (Mattila et al., 2022). A recent meta-analysis of molecular point-of-care testing for acute respiratory tract infections similarly found little to no effect on overall antibiotic use, treatment duration, 30-day mortality, or ICU admission, despite improved prescribing appropriateness (Li et al., 2026). These findings indicate that rapid pathogen identification alone does not necessarily improve patient outcomes. Multiplex PCR panels provide rapid detection of predefined pathogens, whereas mNGS may identify unexpected, off-panel, or mixed organisms but entail higher cost, longer turnaround time, and greater interpretive complexity. Unlike predefined multiplex PCR panels, mNGS can simultaneously survey multiple pathogen classes, enabling detection of unexpected, off-panel, or mixed organisms. This broader coverage may be particularly valuable in critically ill or diagnostically uncertain patients, but entails higher cost, longer turnaround time, and greater interpretive complexity. The rationale explored here is therefore complementary: mNGS provides broad pathogen information, whereas dWBC adds temporal host-response context for structured post-report reassessment. Although this study did not directly compare mNGS with PCR, it identifies a focused strategy for prospective evaluation.

Adding dWBC to the baseline model was associated with numerically higher apparent and optimism-corrected AUCs and lower Brier scores. Nevertheless, the DeLong comparison of the apparent AUCs was not statistically significant (P = 0.088), and paired confidence intervals for optimism-corrected performance differences were not estimated; superiority of the dynamic model is therefore unconfirmed. The corrected calibration slope of 0.732 indicates residual overfitting. Similarly, preservation of the positive dWBC coefficient in 99.8% of bootstrap refits reflects conditional direction stability of the finalized model rather than selection stability across the complete development procedure. The dynamic model included five predictors for only 39 actionable events, corresponding to approximately 7.8 events per predictor. Together with the small complete-case cohort and absence of external validation, these findings support an exploratory interpretation despite the potential relevance of longitudinal clinical indicators reported in previous studies (Fleuren et al., 2020; Jazayeri et al., 2023; Jiang et al., 2023; Sun et al., 2025).

Clinical actionability in this study was a pragmatic endpoint defined by a documented antimicrobial modification attributed to the available mNGS findings. Because treatment escalation accounted for 39 of 58 actionable cases (67.2%), the observed association between dWBC and actionability may have been driven predominantly by escalation decisions and should not be assumed to apply equally across all antimicrobial-modification categories. Actionability was not a measure of treatment appropriateness, patient benefit, or the entire clinical value of mNGS. Some results may influence diagnosis, infection control, exposure management, public-health action, or prognostic communication without leading to antimicrobial modification. For example, a rabies virus result may remain clinically important even when no opportunity for treatment modification exists. These broader management effects were not systematically captured and should be prespecified in future prospective studies.

The median SOFA score of approximately 8 reflects the selected, critically ill population in which mNGS was used in routine practice. Because sepsis is time-sensitive, mNGS may serve as an early adjunct to conventional testing in selected high-risk patients, complementing rather than replacing culture-based diagnostics. Its implementation requires consideration of cost, laboratory infrastructure, interpretive workload, detection of organisms of uncertain relevance, and limited phenotypic susceptibility information, all of which remain important constraints (Lodise et al., 2019; Umpleby et al., 2022). These findings suggest that dWBC may provide contextual information for post-report interpretation among patients with sepsis who have already undergone mNGS. The intended use of the dynamic model is therefore limited to post-report clinical reassessment. Because dWBC becomes available only after mNGS has been ordered and its report has become available, the model cannot identify patients for testing or reduce unnecessary mNGS use. A high predicted probability should not be interpreted as an instruction to modify treatment. Rather, it may identify situations in which the mNGS findings, current antimicrobial coverage, specimen context, and evolving clinical condition warrant structured reassessment.

Several limitations should be acknowledged. First, this was a small, single-center retrospective study. Exclusion of 142 screened patients with incomplete baseline data and restriction of model development to 72 complete cases may have introduced selection bias. Because the broader cohort lacked complete data for all candidate dynamic indicators, the entire variable-selection procedure could not be reassessed. Prospective studies with standardized and complete collection of all candidate indicators are required to evaluate the reproducibility of the dWBC association. Differences in sex and specimen distribution further limit generalizability. Requiring an eligible post-report measurement before antimicrobial modification may also have introduced measurement-availability or survivor bias. The interval between mNGS report availability and the repeat WBC measurement was not standardized, precluding a reliable summary, and variations in this interval could affect the magnitude and comparability of dWBC. Prospective studies should employ pre-specified time points for repeat measurements. Second, retrospective attribution of treatment changes to mNGS cannot establish causality. Although two physicians who had not participated in the original care independently applied prespecified criteria, incomplete documentation, hindsight bias, shared institutional practice, and the absence of a counterfactual could not be eliminated. The present study does not support universal front-door mNGS testing or define a disease-severity threshold for ordering. Prospective comparisons across severity strata are required to determine appropriate testing thresholds and cost-effectiveness. Third, culture and mNGS specimen types were not uniformly matched, and the composite clinical assessment incorporated mNGS findings; diagnostic-performance estimates should therefore be interpreted as exploratory concordance measures. Finally, bootstrap correction evaluated the finalized models without repeating variable selection, potentially underestimating total development optimism. The study did not assess treatment appropriateness, broader management effects, cost-effectiveness, or patient-centered outcomes. Prospective multicenter validation within a predefined post-report pathway is required.

## 5 Conclusion

In this retrospective cohort, mNGS provided a higher descriptive pathogen-detection yield than conventional culture, although detection did not uniformly result in antimicrobial modification. Among the prespecified peri-report dynamic indicators, dWBC was the only variable retained through model development. A less pronounced decline or an increase in WBC was associated with a greater likelihood of subsequent documented mNGS-related antimicrobial modification. dWBC may therefore provide exploratory contextual information during post-report reassessment. However, the model does not establish treatment appropriateness, clinical benefit, improved outcomes, or clinical utility and requires prospective external validation.

## Data Availability

The metagenomic sequencing data generated in this study are deposited in the Genome Sequence Archive (GSA) of the National Genomics Data Center under accession number CRA051109 (https://ngdc.cncb.ac.cn/gsa/browse/CRA051109). The de-identified clinical data underlying the analyses are available from the corresponding author upon reasonable request, subject to institutional and ethical requirements.

## References

[B1] Benoit P. Brazer N. de Lorenzi-Tognon M. Kelly E. Servellita V. Oseguera M. et al. (2024). Seven-year performance of a clinical metagenomic next-generation sequencing test for diagnosis of central nervous system infections. *Nat. Med.* 30, 3522–3533. 10.1038/s41591-024-03275-1 39533109 PMC11645279

[B2] Blauwkamp T. A. Thair S. Rosen M. J. Blair L. Lindner M. Vilfan I. et al. (2019). Analytical and clinical validation of a microbial cell-free DNA sequencing test for infectious disease. *Nat. Microbiol.* 4, 663–674. 10.1038/s41564-018-0349-6 30742071

[B3] Bloos F. Rüddel H. Thomas-Rüddel D. Schwarzkopf D. Pausch C. Harbarth S. et al. (2017). Effect of a multifaceted educational intervention for anti-infectious measures on sepsis mortality: A cluster randomized trial. *Intensive Care Med.* 43, 1602–1612. 10.1007/s00134-017-4782-4 28466151

[B4] Chiu C. Y. Miller S. A. (2019). Clinical metagenomics. *Nat. Rev. Genet.* 20, 341–355. 10.1038/s41576-019-0113-7 30918369 PMC6858796

[B5] Daher M. Iordanov R. Al Mohajer M. Sohail M. R. Staggers K. A. Hamdi A. M. (2024). Clinical utility of metagenomic next-generation sequencing in fever of undetermined origin. *Ther. Adv. Infect. Dis.* 11:20499361241244969. 10.1177/20499361241244969 38645300 PMC11027448

[B6] Duan H. Li X. Mei A. Li P. Liu Y. Li X. et al. (2021). The diagnostic value of metagenomic next-generation sequencing in infectious diseases. *BMC Infect. Dis.* 21:62. 10.1186/s12879-020-05746-5 33435894 PMC7805029

[B7] Evans L. Rhodes A. Alhazzani W. Antonelli M. Coopersmith C. French C. et al. (2021). Surviving sepsis campaign: International guidelines for management of sepsis and septic shock 2021. *Intensive Care Med.* 47, 1181–1247. 10.1007/s00134-021-06506-y 34599691 PMC8486643

[B8] Fleuren L. M. Klausch T. L. T. Zwager C. L. Schoonmade L. Guo T. Roggeveen L. et al. (2020). Machine learning for the prediction of sepsis: A systematic review and meta-analysis of diagnostic test accuracy. *Intensive Care Med.* 46, 383–400. 10.1007/s00134-019-05872-y 31965266 PMC7067741

[B9] Gu W. Miller S. Chiu C. Y. (2019). Clinical metagenomic next-generation sequencing for pathogen detection. *Annu. Rev. Pathol.* 14, 319–338. 10.1146/annurev-pathmechdis-012418-012751 30355154 PMC6345613

[B10] Jazayeri A. Yang C. C. Capan M. (2023). Frequent temporal patterns of physiological and biological biomarkers and their evolution in sepsis. *Artif. Intell. Med.* 143:102576. 10.1016/j.artmed.2023.102576 37673556 PMC10483113

[B11] Jiang Z. Bo L. Wang L. Xie Y. Cao J. Yao Y. et al. (2023). Interpretable machine-learning model for real-time, clustered risk factor analysis of sepsis and septic death in critical care. *Comput. Methods Programs Biomed.* 241:107772. 10.1016/j.cmpb.2023.107772 37657148

[B12] Kadri S. S. Lai Y. L. Warner S. Strich J. Babiker A. Ricotta E. et al. (2021). Inappropriate empirical antibiotic therapy for bloodstream infections based on discordant in-vitro susceptibilities: A retrospective cohort analysis of prevalence, predictors, and mortality risk in US hospitals. *Lancet Infect. Dis.* 21, 241–251. 10.1016/S1473-3099(20)30477-1 32916100 PMC7855478

[B13] Kalantar K. Neyton L. Abdelghany M. Mick E. Jauregui A. Caldera S. et al. (2022). Integrated host-microbe plasma metagenomics for sepsis diagnosis in a prospective cohort of critically ill adults. *Nat. Microbiol.* 7, 1805–1816. 10.1038/s41564-022-01237-2 36266337 PMC9613463

[B14] Kox M. Bauer M. Bos L. D. J. Bouma H. Calandra T. Calfee C. et al. (2026). The immunology of sepsis: Translating new insights into clinical practice. *Nat. Rev. Nephrol.* 22, 30–49. 10.1038/s41581-025-01004-6 40993251

[B15] Kumar A. Roberts D. Wood K. E. Light B. Parrillo J. Sharma S. et al. (2006). Duration of hypotension before initiation of effective antimicrobial therapy is the critical determinant of survival in human septic shock. *Crit. Care Med.* 34, 1589–1596. 10.1097/01.CCM.0000217961.75225.E9 16625125

[B16] Langelier C. Kalantar K. L. Moazed F. Wilson M. Crawford E. D. Deiss T. et al. (2018). Integrating host response and unbiased microbe detection for lower respiratory tract infection diagnosis in critically ill adults. *Proc. Natl. Acad. Sci. U S A.* 115, E12353–E12362. 10.1073/pnas.1809700115 30482864 PMC6310811

[B17] Li Q. Zhou Q. Fan J. Feng X. Lai H. Chen Y. et al. (2026). Impact of molecular point-of-care testing for respiratory pathogens on antibiotic use and clinical outcomes in acute respiratory tract infections: A systematic review and meta-analysis. *EClinicalMedicine* 92:103799. 10.1016/j.eclinm.2026.103799 41732193 PMC12925124

[B18] Lodise T. P. Bonine N. G. Ye J. M. Folse H. Gillard P. (2019). Development of a bedside tool to predict the probability of drug-resistant pathogens among hospitalized adult patients with gram-negative infections. *BMC Infect. Dis.* 19:718. 10.1186/s12879-019-4363-y 31412809 PMC6694572

[B19] Mattila S. Paalanne N. Honkila M. Pokka T. Tapiainen T. (2022). Effect of point-of-care testing for respiratory pathogens on antibiotic use in children: A randomized clinical trial. *JAMA Netw. Open* 5:e2216162. 10.1001/jamanetworkopen.2022.16162 35679047 PMC9185185

[B20] Rhee C. Kadri S. S. Dekker J. P. Danner R. Chen H. Fram D. et al. (2020). Prevalence of antibiotic-resistant pathogens in culture-proven sepsis and outcomes associated with inadequate and broad-spectrum empiric antibiotic use. *JAMA Netw. Open* 3:e202899. 10.1001/jamanetworkopen.2020.2899 32297949 PMC7163409

[B21] Rudd K. E. Johnson S. C. Agesa K. M. Shackelford K. Tsoi D. Kievlan D. et al. (2020). Global, regional, and national sepsis incidence and mortality, 1990–2017: Analysis for the Global Burden of Disease Study. *Lancet* 395, 200–211. 10.1016/S0140-6736(19)32989-7 31954465 PMC6970225

[B22] Seymour C. W. Kennedy J. N. Wang S. Chang C. Elliott C. Xu Z. et al. (2019). Derivation, validation, and potential treatment implications of novel clinical phenotypes for sepsis. *JAMA* 321, 2003–2017. 10.1001/jama.2019.5791 31104070 PMC6537818

[B23] Shankar-Hari M. Phillips G. S. Levy M. L. Seymour C. Liu V. Deutschman C. et al. (2016). Developing a new definition and assessing new clinical criteria for septic shock: For the third international consensus definitions for sepsis and septic shock (Sepsis-3). *JAMA* 315, 775–787. 10.1001/jama.2016.0289 26903336 PMC4910392

[B24] Simner P. J. Miller S. Carroll K. C. (2018). Understanding the promises and hurdles of metagenomic next-generation sequencing as a diagnostic tool for infectious diseases. *Clin. Infect. Dis.* 66, 778–788. 10.1093/cid/cix881 29040428 PMC7108102

[B25] Singer M. Deutschman C. Seymour C. Shankar-Hari M. Annane D. Bauer M. et al. (2016). The third international consensus definitions for sepsis and septic shock (Sepsis-3). *JAMA* 315, 801–810. 10.1001/jama.2016.0287 26903338 PMC4968574

[B26] Stanski N. L. Wong H. R. (2020). Prognostic and predictive enrichment in sepsis. *Nat. Rev. Nephrol.* 16, 20–31. 10.1038/s41581-019-0199-3 31511662 PMC7097452

[B27] Sun L. K. Zhang S. Yang Z. Yang F. Wang Z. Li H. et al. (2022). Clinical application and influencing factor analysis of Metagenomic next-generation sequencing (mNGS) in ICU patients with sepsis. *Front. Cell. Infect. Microbiol.* 12:905132. 10.3389/fcimb.2022.905132 35909965 PMC9326263

[B28] Sun Q. Teng R. Shi Q. Liu Y. Cai X. Yang B. et al. (2025). Clinical implement of probe-capture metagenomics in sepsis patients: A multicentre and prospective study. *Clin. Transl. Med.* 15:e70297. 10.1002/ctm2.70297 40181528 PMC11968419

[B29] Torres L. Pickkers P. van der Poll T. (2022). Sepsis-induced immunosuppression. *Annu. Rev. Physiol.* 84, 157–181. 10.1146/annurev-physiol-061121-040214 34705481

[B30] Umpleby H. Dushianthan A. Catton T. Saeed K. (2022). Antimicrobial stewardship programmes focused on de-escalation: A narrative review of efficacy and risks. *J. Emerg. Crit. Care Med.* 6, 23–23. 10.21037/jeccm-22-6

[B31] Van Nynatten L. R. Bokhary D. Wong M. Y. S. Wang J. Fero H. McChesney C. et al. (2025). Predictive enrichment using biomarkers in studies of critically-ill patients with sepsis: A systematic review. *Crit. Care* 29:504. 10.1186/s13054-025-05684-1 41310736 PMC12659080

[B32] Von Rekowski C. P. Fonseca T. A. H. Araújo R. Calado C. R. C. Bento L. Pinto I. (2026). Blood biomarker trajectories in ICU-directed prediction models - A scoping review. *EPMA J.* 17, 427–455. 10.1007/s13167-026-00456-5 42179466 PMC13190975

[B33] Wilson M. R. Sample H. A. Zorn K. C. Arevalo S. Yu G. Neuhaus J. et al. (2019). Clinical metagenomic sequencing for diagnosis of meningitis and encephalitis. *N. Engl. J. Med.* 380, 2327–2340. 10.1056/NEJMoa1803396 31189036 PMC6764751

[B34] Xu F. Chen C. Lu S. Xue M. Ding H. Song Y. et al. (2025). Impact of metagenomics next-generation sequencing on etiological diagnosis and early outcomes in sepsis. *J. Transl. Med.* 23:394. 10.1186/s12967-025-06332-6 40181443 PMC11969879

[B35] Zhang D. Li X. Wang Y. Zhao Y. Zhang H. (2024). The clinical importance of metagenomic next-generation sequencing in detecting disease-causing microorganisms in cases of sepsis acquired in the community or hospital setting. *Front. Microbiol.* 15:1384166. 10.3389/fmicb.2024.1384166 38686114 PMC11056561

[B36] Zhao Y. Zhang W. Zhang X. (2024). Application of metagenomic next-generation sequencing in the diagnosis of infectious diseases. *Front. Cell. Infect. Microbiol.* 14:1458316. 10.3389/fcimb.2024.1458316 39619659 PMC11604630

