# Supplementary material for: Predicting clinically actionable metagenomic next-generation sequencing in sepsis through dynamic inflammatory trajectories
Source: Front Microbiol. 2026 Oct 20;17:1949602. doi: 10.3389/fmicb.2026.1949602 (PMC13727620; doi:10.3389/fmicb.2026.1949602)
Supplement: Supplementary file 1 [file Table_1.docx]

**Supplementary Materials for**

**Predicting Clinically Actionable Metagenomic Next-Generation Sequencing in Sepsis Through Dynamic Inflammatory Trajectories**

***Running title:*** Dynamic Prediction of mNGS Actionability

Xiaocheng Ding, Peng Bao, Xiaopeng Huang, Rui Xu, Xinyi Tang, Yuzhi Wu, Xiaoli Cui, Yang Lu*

Department of Intensive Care Medicine, Affiliated Hospital of Nantong University, Nantong, Jiangsu 226001, China

***Correspondence:**

Yang Lu

Department of Intensive Care Medicine

Affiliated Hospital of Nantong University

E-mail: [tdftluyang@163.com](mailto:tdftluyang@163.com)

**Table S1. Subclassification and operational definitions of actionable and non-actionable mNGS results.**

**Table S2. Missing-data pattern underlying the complete-case model-development cohort.**

**Table S3. Comparison of the complete-case model-development cohort and excluded eligible patients.**

**Table S4. Conditional bootstrap assessment of the finalized baseline and dynamic prediction models.**

**Table S5. Association between dWBC and clinically actionable mNGS results according to baseline WBC level.**

**Figure S1. Apparent calibration curves of the baseline and dynamic prediction models.** The plots compare grouped observed and predicted probabilities in the 72-patient model-development cohort. The diagonal dotted line represents perfect calibration. These apparent plots are descriptive and were not used to establish superiority of either model.

**Figure S2.** **Apparent and bootstrap-corrected calibration of the baseline and dynamic prediction models.** Calibration curves of the final baseline and dynamic models in the 72-patient complete-case model-development cohort. Internal validation was performed using 1,000 bootstrap resamples. The dashed diagonal line represents perfect calibration. The optimism-corrected calibration slopes were 0.783 for the baseline model and 0.732 for the dynamic model.

**Figure S3. Adjusted association between dWBC and clinically actionable mNGS results stratified by baseline WBC count.** Squares represent adjusted ORs and horizontal lines represent 95% CIs. Subgroup models were adjusted for age, diabetes, and baseline ln (PCT). The likelihood-ratio P for interaction was 0.494.

**Table**

**Table S1. Subclassification and operational definitions of actionable and non-actionable mNGS results.**

| **Group** | **Primary category** | **n (%)** | **Operational definition** |
| --- | --- | --- | --- |
| Actionable | Initiation | 0 (0.0%) | No anti-infective therapy before the report; antibacterial therapy initiated on the basis of mNGS. |
| Actionable | Escalation | 39 (67.2%) | Broader antibacterial coverage or addition/substitution to cover a pathogen newly identified by mNGS. |
| Actionable | De-escalation | 8 (13.8%) | Narrower antibacterial spectrum or partial discontinuation while at least one antibacterial agent was retained. |
| Actionable | Discontinuation | 1 (1.7%) | All antibacterial, antifungal, and antiviral agents were discontinued. |
| Actionable | Antifungal modification | 8 (13.8%) | Antifungal therapy was initiated, discontinued, or changed without a concurrent qualifying antibacterial modification. |
| Actionable | Antiviral modification | 2 (3.4%) | Antiviral therapy was initiated, discontinued, or changed without a concurrent qualifying antibacterial or antifungal modification. |
| Non-actionable | Therapy unchanged | 49 (96.08%) | No antimicrobial modification occurred after report availability within the study assessment window. |
| Non-actionable | Modification not documented as attributable to mNGS | 2 (3.92%) | Therapy changed, but contemporaneous documentation did not support attribution to the mNGS result. |

Each actionable case was assigned one mutually exclusive primary category. When concurrent changes occurred, the change linked to the dominant organism was used; if dominance could not be established, priority was escalation/initiation > discontinuation > de-escalation > antifungal/antiviral modification.

**Table S2. Missing-data pattern underlying the complete-case model-development cohort.**

| **Population** | **Missingness pattern** | **n/N (%)** | **Model implication** |
| --- | --- | --- | --- |
| Initial screening cohort | Excluded before overall analysis | 142/251 | Incomplete essential baseline data |
| Model pattern | Both baseline and post-report observed | 72/109 (66.1%) | Complete paired CRP |

**Table S3. Comparison of the complete-case model-development cohort and excluded eligible patients.**

| Characteristic | Complete cases (n=72) | Excluded (n=37) | P value | SMD |
| --- | --- | --- | --- | --- |
| Age, years | 66.50 [55.50, 75.50] | 66.00 [58.00, 78.00] | 0.718 | -0.135 |
| Male sex | 64 (88.9%) | 10 (27.0%) | <0.001 | 1.253 |
| Hypertension | 34 (47.2%) | 13 (35.1%) | 0.307 | 0.246 |
| Diabetes mellitus | 19 (26.4%) | 9 (24.3%) | 1.000 | 0.047 |
| Coronary heart disease | 8 (11.1%) | 3 (8.1%) | 0.746 | 0.102 |
| Chronic obstructive pulmonary disease | 1 (1.4%) | 0 (0.0%) | 1.000 | 0.167 |
| Septic shock | 15 (20.8%) | 14 (37.8%) | 0.069 | -0.373 |
| Baseline WBC, ×10^9/L | 11.80 [8.66, 17.10] | 11.40 [5.10, 14.20] | 0.306 | 0.156 |
| Baseline neutrophils, % | 88.85 [83.53, 93.10] | 89.40 [84.20, 93.50] | 0.629 | -0.044 |
| Baseline CRP | 110.71 [42.15, 212.98] | 113.02 [43.73, 214.29] (n=32) | 0.980 | 0.014 |
| Baseline PCT | 2.00 [0.62, 13.45] | 5.20 [1.20, 39.00] | 0.106 | -0.376 |
| Baseline SOFA | 8.00 [5.00, 11.00] | 10.00 [5.00, 13.00] | 0.157 | -0.328 |
| Baseline APACHE II | 20.00 [15.50, 25.50] | 22.00 [15.00, 28.00] | 0.365 | -0.152 |
| Antimicrobial exposure before mNGS, days | 4.00 [2.00, 8.00] | 3.00 [2.00, 6.00] | 0.309 | 0.031 |
| dWBC, ×10^9/L | -1.74 [-6.56, 1.37] | -1.70 [-5.90, 3.30] | 0.833 | 0.082 |
| dNEUT, percentage points | -4.80 [-11.32, 1.45] | -3.60 [-9.30, 1.70] | 0.595 | -0.129 |
| dLnPCT | -1.19 [-2.58, 0.00] | -1.14 [-2.94, -0.13] | 0.843 | 0.160 |
| dSOFA | -2.00 [-4.00, -1.00] | -2.00 [-2.00, 1.00] | 0.034 | -0.332 |
| dAPACHE II | -2.00 [-6.00, 0.25] | -1.00 [-3.00, 1.00] | 0.102 | -0.254 |
| Actionable mNGS result | 39 (54.2%) | 19 (51.4%) | 0.841 | 0.056 |
| Hospital stay, days | 19.50 [12.00, 30.25] | 18.00 [9.00, 32.00] | 0.446 | -0.019 |
| ICU stay, days | 15.00 [8.00, 26.25] | 17.00 [8.00, 28.00] | 0.694 | 0.038 |
| Hospitalization cost | 16.20 [9.03, 33.64] | 10.44 [5.32, 17.30] | 0.017 | 0.402 |

Data are median [IQR] or n (%). Continuous variables used the Mann-Whitney U test and categorical variables Fisher's exact test. SMD, standardized mean difference.

**Table S4.** Conditional bootstrap assessment of the finalized baseline and dynamic prediction models.

| Model | Metric | Apparent estimate | Mean optimism/degradation | Optimism-corrected estimate | Corrected 95% CI |
| --- | --- | --- | --- | --- | --- |
| Baseline | AUC | 0.744 | 0.046 | 0.697 | 0.598-0.820 |
| Dynamic | AUC | 0.816 | 0.048 | 0.768 | 0.678-0.868 |
| Baseline | Brier score | 0.199 | 0.030 | 0.228 | 0.183-0.286 |
| Dynamic | Brier score | 0.173 | 0.034 | 0.207 | 0.157-0.272 |
| Baseline | Calibration intercept | 0.000 | - | 0.054 | -0.461-0.533 |
| Dynamic | Calibration intercept | 0.000 | - | 0.076 | -0.412-0.530 |
| Baseline | Calibration slope | 1.000 | 0.217 | 0.783 | 0.442-1.306 |
| Dynamic | Calibration slope | 1.000 | 0.268 | 0.732 | 0.356-1.170 |

For AUC, optimism was defined as performance in the bootstrap resample minus performance in the original cohort, and the corrected estimate was calculated as the apparent AUC minus mean optimism. For the Brier score, degradation was defined as performance in the original cohort minus performance in the bootstrap resample, and the corrected estimate was calculated as the apparent Brier score plus mean degradation. Calibration estimates summarize application of bootstrap-refitted models to the original cohort. Variable selection was not repeated within bootstrap resamples.

**Table S5.** Association between dWBC and clinically actionable mNGS results according to baseline WBC level.

| Baseline WBC subgroup | N | Actionable, n (%) | Adjusted OR for dWBC | 95% CI | P value | P for interaction |
| --- | --- | --- | --- | --- | --- | --- |
| ≤12 × 10^9/L | 37 | 25 (67.6%) | 1.15 | 0.95-1.38 | 0.144 | 0.494 |
| >12 × 10^9/L | 35 | 14 (40.0%) | 1.16 | 1.01-1.34 | 0.034 | - |

Subgroup-specific models were adjusted for age, diabetes, and baseline ln (PCT). The P value for interaction was obtained from a likelihood-ratio comparison of full-cohort models with and without the continuous baseline WBC×dWBC interaction term.

**Figure**

**Figure S1. Apparent calibration curves of the baseline and dynamic prediction models.** The plots compare grouped observed and predicted probabilities in the 72-patient model-development cohort. The diagonal dotted line represents perfect calibration. These apparent plots are descriptive and were not used to establish superiority of either model.

**Figure S2. Apparent and bootstrap-corrected calibration of the baseline and dynamic prediction models.**

**Figure S3. Adjusted association between dWBC and clinically actionable mNGS results stratified by baseline WBC count.**
